# Severe Hypernatremia in a Significantly Underweight Female Child

**DOI:** 10.1155/2021/4061852

**Published:** 2021-11-03

**Authors:** Megan B. Coriell, Prasanthi Gandham, Kupper Wintergerst, Bradly Thrasher

**Affiliations:** ^1^University of Louisville School of Medicine, Louisville, KY, USA; ^2^Norton Children's Hospital, Louisville, KY, USA

## Abstract

In this study, we present the case of a 5-year-old female who presented for evaluation of dehydration with labs that revealed significant hypernatremia concerning for diabetes insipidus (DI). Further evaluation revealed that she had underlying chronic malnutrition. Her diagnostic work up for DI produced some evidence consistent with DI while other data indicated otherwise, bringing up the possibility of partial DI. She was ultimately diagnosed with sporadic vasopressin release secondary to her chronic malnutrition. This case illustrates another effect chronic malnutrition can have on pediatric patients along with the importance of a broad differential for patients with severe hypernatremia.

## 1. Introduction 

It is well known that chronic malnutrition in childhood can have both short-term and long-term health consequences. Malnutrition in childhood not only effects physical growth and brain development but has also been shown to delay puberty [1, 2]. However, there is a paucity of data on the possible effects malnutrition can have on vasopressin and the hypothalamic-pituitary-adrenal axis. In this article, we present the case of a 5-year-old female whose initial chief complaint was dehydration. She had significant hypernatremia concerning for diabetes insipidus (DI) and was found to have underlying chronic malnutrition. This case illustrates another health consequence of chronic malnutrition along with the importance of a broad differential for patients with severe dehydration and hypernatremia.

## 2. Case Presentation

A 5-year-old female presented to the emergency department with a three-day history of poor oral intake, mild upper respiratory symptoms, and concerns for dehydration. Her history was notable for premature birth at 29 weeks, neonatal respiratory distress syndrome, and necrotizing enterocolitis. History was limited as patient's father was present during her medical evaluation, and patient was in mother's care prior to arrival. Of note, parents were separated but still shared custody.

Initial evaluation at an outside facility included a comprehensive metabolic panel (CMP), which revealed a sodium of 176 mmol/L. She was given a lactated Ringer's bolus, resulting in a decrease of her sodium to 171 mmol/L.  Table 1 provides the initial lab results. Other testing included a rapid strep test, chest X-ray, complete blood count, and influenza and COVID-19 testing, all of which were unremarkable. A urinalysis was notable for specific gravity 1.020, ketones 5 mg/dL, protein 500 mg/dL, nitrite negative, leukocyte esterase 100, and white blood cells 10–15 HPF. She was then transferred to Norton Children's Hospital for further management of hypernatremia. During transport, the patient reported hunger and stated that she was not given food at home.

On arrival to our facility, a basic metabolic panel (BMP) was repeated which was notable for sodium 158 mmol/L, potassium 2.7 mmol/L, chloride 119 mmol/L, carbon dioxide 32 mmol/L, and glucose 108 mg/dL. Random urine sodium and random urine creatinine were within normal limits. Random urine osmolality was decreased at 99.5 mOsm/kg, and serum osmolality was increased at 330 mOsm/kg. Given these findings, pediatric endocrinology was consulted due to hypernatremia and concern for DI. Additional labs showed a low prealbumin of 8 mg/dL (reference range: 11.0–23.0 mg/dL), normal renin activity of 4.5 ng/mL/hr, low aldosterone of <3.0 ng/dL (reference range 4.0–44.0 ng/dL), and normal AM cortisol of 13.7 ug/dL. She was started on normal saline fluids, given oral potassium-chloride replacement, and admitted to the pediatric ICU for further management.

On day 2 of admission, her sodium level trended down to 155 mmol/L with fluid resuscitation and her appetite improved. However, her urine output (UOP) was significantly elevated at 8.28 mL/kg/hr. Dad denied any history of polyuria or polydipsia. She was toilet trained and had no history of accidents. She had been meeting developmental milestones and doing well in virtual kindergarten.

Her weight on admission was 15.1 kg (1.8^th^ percentile), height was 106 cm (8.9^th^ percentile), and BMI was 13.44 kg/m^2^ (4.8^th^ percentile). Of note, at 2 years of age, her height was in the 71^st^ percentile and weight in the 30^th^ percentile. Given these findings along with her significant electrolyte abnormalities on presentation, forensics was consulted due to concerns for chronic malnutrition. Additional work up included thyroid studies, which showed a thyroid stimulating hormone (TSH) of 6.050 IU/mL (reference range: 0.470–4.680 IU/mL) and free thyroxine (T4) of 1.49 ng/dL (reference range: 0.78–2.19 ng/dL). Her celiac panel was negative, and growth factors were normal for age.

Our patient's sodium level improved with IV fluids and oral intake. Her hypokalemia and alkalosis also improved with potassium-chloride replacement. She was noted to have a mild acute kidney injury on admission with an elevated creatinine of 0.77 mg/dL, but her creatinine level normalized quickly with rehydration. Although clinical evidence of dehydration resolved, including physical examination findings and normalization of her heart rate and blood pressure, her sodium level rose to 149–150 mmol/L each time IV fluids were weaned. She also continued to have polyuria, although this slowly improved throughout admission. Due to the persistent hypernatremia and polyuria, DI continued to be a concern, and a modified water deprivation test was performed. During a traditional water deprivation test, the patient is made NPO, and serial labs are checked every 1-2 hours. These labs include serum sodium, serum osmolality, urine osmolality, and urine specific gravity. If the patient has DI, the urine osmolality and specific gravity will remain low despite a high serum osmolality and hypernatremia [3]. In our patient's case, she was made NPO at midnight, and fasting AM labs were obtained. Initial labs were concerning for DI with a low urine osmolality in the setting of a high serum sodium and serum osmolality. Desmopressin (DDAVP) was ordered to determine if administration would lead to concentration of urine. This would differentiate central from nephrogenic DI. However, before DDAVP could be given, father allowed the patient to drink. Of note, her UOP had decreased significantly, and so the decision was made to monitor. Another water deprivation test was performed overnight, and results were not consistent with DI. The lab results from her water deprivation tests are given in Table 2.

While these mixed results were not consistent with a frank diagnosis of complete vasopressin deficiency, the possibility of a partial DI diagnosis was considered. A pituitary MRI with and without contrast was obtained to look for abnormalities that may explain her findings. This showed a normal pituitary gland with decreased bright spot on T1 weighted imaging, likely within range of normal. This decrease in the bright spot has been reported in patients with DI but is also a normal variant found in 10% of the general population [4].

At discharge, our patient's sodium level had remained within normal range for several days off IV fluids and despite fasting for prolonged periods of time. Her weight increased from 15.1 kg to 16.3 kg during admission. She was discharged in the care of her father per child protective services with a plan for close follow-up.

She was seen by pediatric endocrinology two weeks after hospital discharge, and her father reported no signs of excessive urination or excessive thirst. Her weight was up to 17.3 kg from 16.3 kg at discharge. A BMP, thyroid function studies, and repeat growth factors were all within normal limits. At her endocrinology follow-up three months later, a repeat CMP was still normal, and she had no symptoms of polyuria or polydipsia. She continued to have appropriate weight gain. Based on her course, findings, and resolution of symptoms with adequate weight gain, she was diagnosed with hypernatremia and dehydration related to sporadic vasopressin release secondary to her malnutrition.

## 3. Discussion

The differential for severe hypernatremia in this clinical vignette included DI, hypernatremic dehydration, salt intoxication, primary hyperaldosteronism, and Gitelman/Bartter syndrome. The diagnostic criteria for DI includes polydipsia and polyuria in the setting of hypernatremia, serum osmolality >300 mOsm/kg, urine osmolality <300 mOsm/kg, and urine specific gravity <1.010 [4, 5]. Polyuria is defined as > 100–110 mL/kg/day in children ≤2 years of age and >50 mL/kg/day in older children [5]. DI was high on the differential given our patient's low urine osmolality and low urine specific gravity in the setting of a high serum osmolality and hypernatremia. She also had significant polyuria during the first 24 hours of admission with a UOP of 8.28 mL/kg/hr. However, there was no history of polyuria prior to admission. Later during admission, labs were no longer consistent with DI. This brought up the possibility of partial DI. Patients with partial DI have a partial deficiency of or partial response to vasopressin. They can typically somewhat concentrate their urine, with urine osmolality between 300 and 800 mOsm/kg following a water deprivation test. They typically have a <50% increase in urine osmolality after administration of DDAVP as compared to a >50% increase in urine osmolality in patients with complete DI [3, 5, 6].

Hypernatremic dehydration was also high on the differential given her significant dehydration and concerns for malnutrition on presentation. Hypernatremic dehydration occurs when water loss is more than solute loss and serum sodium is > 150 mmol/L [7]. The history of very poor oral intake for 2-3 days in the setting of a possible illness further supports this diagnosis. If this was our patient's diagnosis, we would anticipate her UOP to slowly return to baseline as her hydration status improved.

Salt intoxication usually involves a random urine sodium of >25 mmol/L and a fraction of excreted sodium (FENa) of >2%. Patients also typically have altered mental status (AMS) due to the acute change in their serum sodium level [8]. This diagnosis was less likely in our patient given her initial random urine sodium of 23 mmol/L, FENa of 2%, and no episodes of AMS before or during admission.

Primary hyperaldosteronism presents with hypernatremia and hypokalemia. This is a very rare condition, and patients typically have hypertension and mild volume expansion. Our patient had no hypertensive episodes and was dehydrated, as opposed to volume expanded, on presentation. Her renin and aldosterone were both appropriately suppressed in the setting of hypernatremia and hypokalemia, ruling out primary hyperaldosteronism.

Our patent's history of poor growth over the past several months was concerning for long standing malnutrition. Her thyroid studies showed a slightly elevated TSH and a normal free T4. The patient was clinically euthyroid. These lab findings were most likely due to her acute presentation. Her short stature was likely secondary to chronic malnutrition as growth factors were normal for age.

After narrowing down our differential, we found that no diagnosis completely fit our patient's case. We then turned to the literature to look for similar cases. Review of the literature revealed case reports of abnormal arginine vasopressin (AVP) axis function in patients with anorexia nervosa (AN). It is hypothesized that refeeding patients with severe malnutrition secondary to AN led to the development of DI [9]. These patients' AVP axes recovered over time, usually over the course of two weeks to several months [9, 10]. Several studies have shown alterations of osmoregulation in patients with AN, which is thought to be associated with severity of and duration of AN [11]. Additionally, it has been noted that these patients tend to have sporadic release of vasopressin [10]. Sporadic release of vasopressin could lead to a combination of both normal labs and labs consistent with DI. It is possible that our patient was experiencing a similar phenomenon occurring secondary to her chronic malnutrition. Our patient experienced complete resolution of her symptoms and lab abnormalities with appropriate nutrition and weight gain.

## 4. Conclusion

This case illustrates that severe electrolyte abnormalities can be seen in the setting of severe/prolonged malnutrition, and thus malnutrition should be considered in these cases. As demonstrated in patients with AN, severe/prolonged malnutrition can cause sporadic vasopressin release, leading to labs consistent with partial DI. In cases of sporadic vasopressin release secondary to malnutrition, adequate healthy weight gain will result in normalization of vasopressin function and, ultimately, resolution of symptoms.

## Figures and Tables

**Table 1:** Initial electrolytes obtained at outside facility.

Lab	Initial CMP	BMP after fluid bolus	Reference ranges (adult ranges)
Sodium (mmol/L)	176 (H)	171 (H)	136–145 mmol/L
Potassium (mmol/L)	3.38 (L)	3.20 (L)	3.5–5 mmol/L
Chloride (mmol/L)	137 (H)	135 (H)	98–107 mmol/L
Carbon dioxide (mmol/L)	26	25	21–31 mmol/L
Glucose (mg/dL)	165 (H)	168 (H)	80–100 mg/dL
BUN (mg/dL)	33 (H)	33 (H)	7–25 mg/dL
Creatine (mg/dL)	0.77	0.79	0.6–1.3 mg/dL
Total protein (g/dL)	7.2		6.4–8.9 g/dL
Albumin (g/dL)	4.60		3.5–5.7 g/dL
Calcium (mg/dL)	9.5	9.3	8.6–10.8 mg/dL
AST (IU/L)	26		13–35 IU/L
ALT (IU/L)	24		7–52 IU/L
Alkaline phosphatase (IU/L)	91		38–104 IU/L
Total bilirubin (mg/dL)	0.5		0.3–1.0 mg/dL

**Table 2:** Water deprivation test results.

Lab	Test 1				Test 2	
	11/11/20 at 2157	11/12/20 at 0544	11/12/20 at 0845	11/12/20 at 1150	11/13/20 at 0629	11/13/20 at 1135
Serum sodium (mmol/L)	145	147 (H)	139	140	139	140
Serum osmolality (mOsm/kg)	305 (H)	311(H)	296	305	298	300
Urine sodium (mmol/L)	27	34	186	189	40	
Urine osmolality (mOsm/kg)	101.5 (L)	221 (L)	504	518.5	253.5 (L)	255.5 (L)
Specific gravity		1.006	1.012	1.011	1.008	1.010

## Data Availability

This is a case report of a single patient; to protect privacy and respect confidentiality, none of the raw data have been made available in any public repository. The original reports, laboratory studies, imaging studies, and outpatient clinic records are retained as per normal procedure within the medical records of our institution.
